# Animal models of enterovirus 71 infection: applications and limitations

**DOI:** 10.1186/1423-0127-21-31

**Published:** 2014-04-17

**Authors:** Ya-Fang Wang, Chun-Keung Yu

**Affiliations:** 1 Center of Infectious Disease and Signaling Research, Collage of Medicine, National Cheng Kung University, Tainan, Taiwan; 2 National Institute of Infectious Diseases and Vaccinology, National Health Research Institutes, Zhunan, Taiwan; 3 Department of Microbiology and Immunology, Collage of Medicine, National Cheng Kung University, No. 1, University Road, Tainan 70101, Taiwan; 4 National Laboratory Animal Center, National Applied Research Laboratories, Taipei, Taiwan

**Keywords:** Enterovirus 71, Animal models, Pathogenesis

## Abstract

Human enterovirus 71 (EV71) has emerged as a neuroinvasive virus that is responsible for several outbreaks in the Asia-Pacific region over the past 15 years. Appropriate animal models are needed to understand EV71 neuropathogenesis better and to facilitate the development of effective vaccines and drugs. Non-human primate models have been used to characterize and evaluate the neurovirulence of EV71 after the early outbreaks in late 1990s. However, these models were not suitable for assessing the neurovirulence level of the virus and were associated with ethical and economic difficulties in terms of broad application. Several strategies have been applied to develop mouse models of EV71 infection, including strategies that employ virus adaption and immunodeficient hosts. Although these mouse models do not closely mimic human disease, they have been applied to determine the pathogenesis of and treatment and prevention of the disease. EV71 receptor-transgenic mouse models have recently been developed and have significantly advanced our understanding of the biological features of the virus and the host-parasite interactions. Overall, each of these models has advantages and disadvantages, and these models are differentially suited for studies of EV71 pathogenesis and/or the pre-clinical testing of antiviral drugs and vaccines. In this paper, we review the characteristics, applications and limitation of these EV71 animal models, including non-human primate and mouse models.

## Review

### Introduction

Since the effective control of poliovirus, enterovirus 71 (EV71) has been regarded as the most important neurotropic enterovirus. EV71 belongs to the species *Human enterovirus A* of the genus *Enterovirus* within the family *Picornaviridae* and is the major pathogen of hand-foot-mouth disease (HFMD). EV71 was first described in 1974 after it was isolated from patients in California and from then on, EV71 infection has been reported in at least 12 small and large outbreaks throughout the world [1-4]. Two subsequent outbreaks occurred in Malaysia and Taiwan in the late 1990s, and these outbreaks mainly affected young children and were characteristically associated with severe neurological complications and high rates of fatality [3,5]. Since then, the Asia-Pacific region, including Taiwan, Malaysia, Vietnam, Singapore, Japan and China, has experienced more frequent large-scale EV71-associated HFMD epidemics.

EV71 infection is generally mild and self-limited, but occasionally such infection leads to central nervous system (CNS) infections that include aseptic meningitis, brainstem encephalitis and acute flaccid paralysis [6,7]. Fatal EV71 cases develop and progress rapidly and are typically associated with neural complications, pulmonary edema and function collapse, which make clinical management challenging [8,9]. The precise mechanisms of EV71-mediate disease, particularly the neuropathogenesis, are still not fully understood because suitable and relevant animal models have not been established.

Similar to poliovirus, EV71 has a limited host range; humans are the only known natural host. Early studies showed that the members of *Picornaviridae* display distinct pathogenicities in newborn mice for which neither poliovirus nor echovirus are pathogenic; coxsackie A viruses were found to cause generalized myositis, and coxsackie B viruses were found to induce myocarditis [10,11]. Soon after the discovery of EV71, experimental EV71 infections were reported in neonatal mice [12,13] and cynomolgus monkeys [14]. However, EV71 experimental infection models were not established until the severe outbreaks in the Asia-Pacific region around the late 1990s [6,15].

Experimental models of EV71 infection, including murine and non-human primate models, have been developed as alternative means to determine the pathogenesis of and treatments and preventions for the diseases caused by this virus. Indeed, works based on these models have advanced our knowledge of EV71 infection and disease, and most importantly, have accelerated the development of EV71 vaccines. In this review, we discuss these animal models and emphasize their applications and limitations (summarized in Table 1).

**Table 1:** Enterovirus 71 animal models for pathogenesis studies

**Animal**	**Virus strain**	**Infection route/dose**	**Clinical manifestation of animals and viral replication sites**	**Ref. No.**
Non-human primates				
Cynomolgus monkey	Clinical isolates and prototype BrCr	i.s./10^6^ CCID_50_	The monkeys developed neurological manifestations including both pyramidal and extrapyramidal tract signs, such as flaccid paralysis, tremor and ataxia. The virus replicated in the spinal cord, brainstem, cerebella and cerebrum. EV71 had a wider neurotropism than that of polioviruses.	[19,20,27,28]
		i.v./10^5.5^ to 10^7^ TCID_50_		
Rhesus monkey, adult and neonate	Clinical isolate (FY-23, C4 genotype)	i.c., i.v./10^6.5^ CCID_50_	The infected adult monkeys developed CNS infection and neuron impairment with extra-neuronal pathological changes confined to the lung tissues and not the pancreas and spleen, where high viral loads were detected.	[2,29,30]
		i.t./ 10^4.5^ CCID_50_	In neonate monkeys, HFMD-liked papules and vesicles were found on the limbs and in the mouth after intratracheal infection. However, the typical neurological complication was not observed. High viral titers were transiently detected in the brown adipose tissue, skeletal muscle and CNS.	
		oral/10^6.5^ CCID_50_ twice		
Mice - immunocompetent				
ICR, 1- to 14-day-old	Mouse-adapted EV71 strain (MP4, C2 genotype)	oral/5 × 10^6^ pfu	The mice developed rear limb paralysis (with massive and widespread necrotizing myositis) and neuropathologies (with neuronal loss and apoptosis) in the spinal cord and brainstem before death. The spinal cord, brain and muscle were the major organs for virus replication in the late phase of infection. Retrograde axonal transport in neurons might represent the major transmission route of EV71 in mice.	[7,13,36,38,39]
		i.m./5 × 10^3^ pfu		
		i.c./5 × 10^4^ pfu		
BALB/c, 1- to 7-day-old	Mouse-adapted strain (MP-26 M)	i.c. /3.4 × 10^4^ TCID_50_	The mice developed limb paralysis followed by death after MP-26 M infection. Skeletal muscle displayed severe necrotizing myositis and contained a high viral load. Viruses were also isolated from the blood, hearts, livers, spleens and brains of infected mice. The VP1 mutation (G145E) alone was sufficient to increase the virulence of the virus in mice.	[33]
		i.m./3.4 × 10^3^ TCID_50_		
		i.p./3.4 × 10^4^ TCID_50_		
ICR, 2-week-old	Mouse-adapted strain (MAVs, B3 genotype)	i.c., i.p., s.c., oral/10^5^ CCID_50_	The mice developed paralysis followed by death after i.c., i.p., s.c. and i.m. administration but not after oral inoculation of the virus. The highest viral titers were detected in the skeletal muscle, spleen and spinal cord. The virus might enter the CNS via peripheral motor nerves after skeletal muscle infection.	[37]
		i.m./3 × 10^5^ CCID_50_		
ICR, 1-day-old	Mouse muscle-adapted strain (Fuyang-0805a, C4 genotype)	i.p./10^5^ TCID_50_	The Fuyang-0805a strain showed strong myotropism and induced severe necrotizing myositis in both skeletal and cardiac muscles. The virus was detected in the muscle, heart and intestines.	[42]
Mice - immunodeficiency				
NOD/SCID, 3- to 4-week old	NOD/SCID mouse-adapted strain	i.c./10^6^ CCID_50_	The infected mice showed paralysis of the hind limbs. Viral RNA was first detected in the CNS and serum and then high copy numbers were detected in the heart, skeletal muscle and spinal cord.	[34]
	(EV71(NOD/SCID), B1 genotype)			
AG129, 10-week-old	A129 and AG129 mouse-adapted strain (B2 genotype)	i.p./5.2 × 10^4^ TCID_50_	The infected AG129 mice but not A129 mice developed limb paralysis, eye irritation, loss of balance and control of movements and exhibited high mortality.	[43]
AG129, 2-week-old	Clinical isolate (5865/SIN/00009, B4 genotype)	i.p./10^6^ pfu	The infected mice displayed progressive limb paralysis before death. The virus accumulated in the CNS and resulted in massive damage in the limb muscles, brainstem and anterior horn of spinal cord. Low viral particles in the limbs after oral inoculation indicated that the paralysis was a consequence of EV71 neuroinvasion.	[32]
		oral/10^7^ pfu		
Gerbils, 21-day-old	Clinical isolate (EV71/58301, C4 genotype)	i.p./10^5^ TCID_50_	The infected animals developed neurological disorders such as hind limb paralysis, slowness, and ataxia before death. Significantly high viral titers were detected in the spinal cord, brainstem and skeletal muscle.	[44]
Transgenic mice				
PSGL-1, 10-day-old	Clinical isolates C4 genotype and Mouse muscle-adapted strain (Fuyang-0805a, C4 genotype)	i.p./10^8^ TCID_50_	The transgenic mice were only susceptible to a mouse muscle-adapted EV71 strain and not the EV71 clinical isolates and exhibited severe symptoms that were comparable to those of the wild-type mice upon EV71 infection. High viral titers were detected in the muscle, spinal cord and brain after mouse-adapted virus infection. This study concluded that human PSGL-1 alone was not sufficient to provoke the infectivity of EV71 in mice.	[50]
SCARB2, 1- to 14-day-old	Clinical isolates, C2, C4, B4 and B5 genotypes and CA16	s.c./3 × 10^4^ to 10^6^ pfu	EV71 B genotypes were capable of inducing HFMD-like lesions and neurological disease with limb paralysis in 1-day-old but not transgenic mice older than 2 weeks of age. In contrast, 7- and 14-day-old but not 21-day-old transgenic mice were more susceptible to the C genotypes of EV71 and coxsackievirus A16 and exhibited more severe CNS diseases, limb paralysis and death than did the non-transgenic mice.	[52]
SCARB2, 3-week-old	Clinical isolates (Isehara/Japan/99, C genotype)	i.c., i.v., i.p., oral/ 10^4^ to 10^6^ TCID_50_	The transgenic mice displayed ataxia, paralysis and death. The CNS, including the spinal cord, brainstem, cerebellum, cerebrum, hypothalamus and thalamus, was the major replication site for the virus.	[35]

*i.s.*: intraspinal; *i.v.*: intravenous; *i.c.*: intracranial/intracerebral; *i.t.*: intratracheally; *i.p.*: intraperitoneal; *s.c.*: subcutaneous.

### Clinical features of EV71 infection in humans

Like other types of enteroviral infections, EV71 infection may cause persistent fever, diarrhea, rashes, aseptic meningitis and encephalitis, usually without life-threatening manifestations [3,16]. The majority of infected children have asymptomatic and self-limiting infections. Mild cases are characterized by upper respiratory tract infection and cutaneous diseases such as HFMD and herpangina. The illness is characterized by 3–4 days of fever and the development of a vesicular enanthem on the hands, feet and buttocks and herpangina that involves popular lesions on the mucosa of the anterior pillars of Fauces [7]. However, potentially life-threatening neurological and systemic complications, such as brain stem encephalitis, acute flaccid paralysis, autonomic nervous system dysregulation and pulmonary edema, are of the greatest clinical and public concern [3,17-20].

### Animal models used for the study of EV71 tropism and pathogenesis

#### a. Non-human primate models

Early studies showed that non-human primates, including cynomolgus, rhesus and green monkeys, are susceptible to EV71 infection [12,14,21]. Non-human primate models have been used to evaluate and characterize the neurovirulence of EV71 since the outbreaks of the late 1990s. Similar to the neurological manifestations in humans, cynomolgus monkeys display both pyramidal tract signs (flaccid paralysis) and extrapyramidal tract signs (including tremor and ataxia) with a broad viral antigen distribution that involves the spinal cord, brainstem, cerebellar cortex, dentate nuclei and cerebrum following intraspinal and intravenous inoculation of EV71 [22,23]. The neuropathological features are highly consistent with those observed in humans with severe EV71 encephalitis at autopsy [5,18,24-26], which is indicative of the similarity of the susceptibilities of human and cynomolgus monkey CNS tissues to EV71. EV71 exhibits a wider neurotropism than does poliovirus in cynomolgus monkeys, and wild-type strains, including those isolated from patients with fatal encephalitis or hand, foot, and mouth disease (HFMD), exhibit no marked differences with respect to neurovirulence after infection; thus, this monkey species may not be suitable for assessing the neurovirulence level of the virus [22,23]. Additionally, the clinical manifestations in cynomolgus monkeys are not correlated with those in patients. The monkeys do not manifest cutaneous lesions or develop pulmonary edema, although they have brainstem lesions [22,23]. Pulmonary edema has been reported to be frequently associated with fatal EV71 infection in children and is considered to be related to the damage to the brainstem [4,5,27,28].

Cynomolgus monkeys have been used to identify the molecular determinants of EV71. EV71 mutant strains derived from the prototype BrCr strain, which contains mutations in the 5′ non-translated region (NTR), 3D polymerase and 3′ NTR, exhibit attenuated neurovirulence with a limited viral spread in the CNSs of monkeys [29]. Furthermore, this attenuated EV71 strain may be an effective vaccine as it is able to induce a broad spectrum of neutralizing antibody responses against different genotypes of EV71 [30].

Zhang *et al.*[31] reported another non-human primate model involving adult rhesus monkeys. After inoculation with an EV71 clinical isolate (EV71/FY-23) intracerebrally, intravenously, orally (in drinking water) or intratracheally, monkeys develop CNS infections and neuronal impairment with extra-neuronal pathological changes that are confined to the lung tissues (i.e., cellular infiltration and tissue damage) and are not present in the pancreas and spleen where high viral loads are detected. Additionally, the infected-animals do not develop vesicular lesions on the skin and exhibit neither reduced muscle tension in the limbs nor typical neurological symptoms. These results suggest that, in addition to neurotropism, EV71 also elicits respiratory tract tropism in rhesus monkeys; these observations contrast with those based on cynomolgus monkeys and mice. This adult rhesus monkey model presents with all of the infectious and pathogenic processes of a systemic EV71 infection and reveals the clinical manifestations, kinetics of viremia, viral loads in the neuronal and extra-neuronal tissues, immune responses and histopathological changes. Interestingly, intracerebral inoculation induces pulmonary edema and hemorrhages around the hilum of the lung in half of the monkeys. Although it is not known whether the pulmonary edema results from a viral cytolytic effect in the lungs or is a consequence of CNS damage and inflammatory reactions, this adult rhesus monkey model is the first system to show that human EV71 can induce pulmonary edema in a non-natural host.

Hand, foot, and mouth disease-like papules and vesicles are formed on the limbs and in the mouths of 1- to 1.5-month-old neonatal rhesus monkeys after EV71 infection through the intratracheal route [9]. Both the brown adipose tissue and skeletal muscle of the infected monkeys contain large amounts of virus, which suggests that these tissues are the primary target sites of viral replication. Similar to that observed in adult rhesus monkeys, the major inflammatory response is found in the tracheas and lungs of the neonates. In contrast to the cynomolgus monkeys, the typical neurological complications, including flaccid paralysis and ataxia, are not observed in the neonates, although both the medulla oblongata and thalamus contain very high virus titers and display neuropathological lesions. Additionally, the monkeys do not display the clinical symptoms (i.e., serious encephalomyelitis and respiratory failure) that are frequently present in severe human cases. Neonatal rhesus monkeys seem to be a relevant model for EV71-induced HFMD. Subsequent studies have shown that this neonatal monkey model was feasible for evaluating the efficacy of an inactivated EV71 vaccine [32].

#### b. Mouse models

##### b.1. Mouse-adaptation models

In agreement with early studies [12,13], we confirmed that neonatal mice are susceptible to an EV71 clinical isolate (EV71/Tainan/4643/98, GenBank accession number AF304458) [33]. In the course of the development of a mouse model for EV71, we realized that the mice exhibited an age-dependent susceptibility to EV71 infection. Mice older than 14 days of age were resistant to infection with EV71 clinical isolates regardless of the inoculation route. Specifically, neither symptoms nor mortality were observed in the 3- to 6-week-old BALB/c, C3H/HeN or ICR mice after inoculation with the EV7/4643 strain (4.7 × 10^9^ pfu/ml; via through intracranial, intravenous, intraperitoneal, or oral routes; Y. F. Wang and C. K. Yu, unpublished observation). This age-dependent susceptibility to EV71 has been subsequently confirmed in numerous mouse models [34-37].

To increase the virulence of EV71 in mice and to develop an oral infection model that is suitable for the study of the pathogenesis of this disease and for vaccine development, particularly live attenuated vaccines, we generated a mouse–adapted EV71 strain, i.e., EV71/MP4, after four serial passages of the parental strain EV71/4643 in mice. Strain MP4 (5 × 10^6^ PFU/mouse) was capable of orally infecting 7-day-old mice and resulted in rear limb paralysis (with massive and widespread necrotizing myositis) and neuropathology (with neuronal loss and apoptosis) in the spinal cord and brainstem prior to the death of the infected mice [6,15]. Subsequent study [38] and our unpublished observations demonstrated that the mouse-adapted EV71/MP4 strain displayed strong neurotropism and was capable of consistently infecting and producing diseases that included brain infection, flaccid paralysis, pulmonary dysfunction and death in mice up to 14 days of age following intraperitoneal, intramuscular, intracranial, or oral inoculation. Furthermore, by observing the lag time of the disease progression between the distal and proximal viral inoculations via the use of colchicine, a fast axonal transport inhibitor, we demonstrated that retrograde axonal transport in neuron cells, but not hematogenous transport, might be the major transmission route of EV71 in mice [38]. This assumption has been supported by other studies that have used both mice [34,39] and rhesus monkeys [9].

This mouse-adapted EV71 model also allowed us to discover that type I interferon is an essential innate defense mechanism that controls EV71 infection in mice [40] and that EV71 per se inhibited the type I IFN system via the 3C protease while provoking a proinflammatory cytokine response [41].

The mouse-adaption approach has been extensively adopted by other investigators to develop infectious models. Chua *et al.*[35] selected a mouse-adapted strain, i.e., MP-26 M, after six passages of a cell-adapted clinical isolate (CHO-adapted EV71-26 M) in newborn BALB/c mice via intracranial inoculation. After intramuscular, intracerebral or intraperitoneal challenge of the EV71/MP-26 M, both 1- and 7-day-old BALB/c mice developed limb paralysis with high tissue viral loads in the skeletal muscle. Interestingly, this study again demonstrated that the VP1 mutation (G145E) alone was sufficient to increase the virulence of this virus in mice. Our related studies revealed that the substitution of three of the nucleotides of EV71/MP4 in the 5′-NTR (C158U), VP1 (G145E), and VP2 (K149M) regions were responsible for the increased viral infectivity in vitro and the mouse virulence [6,42,43]. These results agree with those of Arita *et al.*[36] and Chua *et al.*[35].

Similarly, Ong *et al.*[39] observed that EV71 could induce encephalomyelitis in 2-week-old but not 4-week-old ICR mice after inoculation with a mouse-adapted strain that was isolated from 1-day-old mice after serial passage of the virus in the brain tissue. The mouse-adapted EV71 strain was capable of infecting mice through both oral and parenteral routes and was capable of entering the CNS via peripheral motor nerves.

Wang *et al.*[44] isolated a mouse muscle-adapted EV71 strain named Fuyang-0805a after four passages of the parental EV71 strain in skeletal muscle. The Fuyang-0805a strain exhibited strong myotropism and induced severe necrotizing myositis in both skeletal and cardiac muscles and induced intestinitis in 1-day-old ICR mice after intraperitoneal infection. There were many mutations in the genome of the muscle-adapted virus, including VP1 (E145Q), which had previously been reported by Chua *et al.*[35] and Huang *et al.*[43].

Arita *et al.*[36] took another approach to generating mouse-adapted EV71 strains in adult immunodeficient mice, which theoretically involved less selective pressure on the virus. The EV71(NOD/SCID) strain was isolated after three passages of the parental virus in the brains of 3-week-old NOD/SCID mice and caused paralysis in 3- to 4-week-old NOD/SCID mice. The adapted virus contained a single amino acid substitution in the VP1 region of EV71 (G145E) that was found to be essential for the mouse-adapted phenotype in NOD/SCID mice. Likewise, Caine *et al.*[45] generated mouse-adapted EV71 strains in adult immunodeficient A129 (α/β IFN receptor deficient) and AG129 (both α/β and γ IFN receptor deficient) mice. The resulting virus was highly lethal to 10-week-old AG129 mice and exhibited 100% lethality; the animals developed clinical symptoms that included limb paralysis, eye irritation and loss of balance before death. It would be worthwhile to know whether this adapted virus also acquired increased virulence for immunocompetent mice.

To overcome the bias of the natural tropism of mouse-adapted/muscle-adapted strains, Khong *et al.*[34] successfully infected 2-week-old AG129 mice with a non-mouse adapted EV71 strain (a clinical isolate termed 5865/SIN/00009). In these immunodeficient mice, the virus exhibited strong neurotropism and induced neurological manifestations after intraperitoneal and oral routes of inoculation. Basically, this model exhibited features that closely resemble those of the murine models that had previously been reported, including clinical manifestations, tissue tropism, and histopathological changes. Collectively, the defect of the IFN system may debilitate the application of this model for the study of disease mechanisms, as alpha IFN is an essential innate defense mechanism for controlling EV71 in mice [40], and defects in beta IFN signaling (type II IFN) may alter the EV71-induced immunopathogenesis observed in immunocompetent hosts [41].

Yao *et al.*[46] experimentally infected 21-day-old gerbils (*Meriones unguiculatus)* with an EV71 clinical isolate (EV71/58301, C4 genotype) via intraperitoneal inoculation. The infected animals developed neurological disorders and histopathological abnormalities that were similar to those that have been reported in the mouse models.

##### b.2. Transgenic mouse models

Viral receptors determine the host ranges and tissue-specific tropisms of enterovirus [47]. Two human receptors for EV71 were identified in 2009: the human P-selectin glycoprotein ligand-1 (PSGL-1, CD162) [48], and the human scavenger receptor class B, member 2 (SCARB2) [49]. PSGL-1 is a sialomucin membrane protein that is expressed exclusively by myeloid and lymphoid white blood cells and platelets and has a major role in the early stages of inflammation. SCARB2 is also known as lysosomal integral membrane protein II or CD36b-like-2. SCARB2 is localized mainly to lysosomes and is widely expressed in many human tissues and cell types. Additionally, sialyated glycans [50] and annexin II [51] have also been reported to be candidate receptors for EV71. Given the success of poliovirus receptor transgenic mice in studies of poliovirus pathogenesis and vaccine efficacy, transgenic mice expressing EV71 receptors have been actively developed.

Liu *et al.*[52] established a transgenic mouse line that expresses the human PSGL-1 gene. However, these animals are only susceptible to a mouse muscle-adapted EV71 strain and not to EV71 clinical isolates, and these animals exhibited severe symptoms that are comparable to those of wild-type mice upon EV71 infection. Given that PSGL-1 is expressed by the dendritic cells in lymph nodes and macrophages in the intestinal mucosa [53], which are the primary sites of EV71 replication, it has been speculated that human PSGL-1 might act as a cofactor in the early stage of EV71 infection and that human PSGL-1 alone is not sufficient to provoke the infectivity of EV71 in mice. Indeed, clinical isolates have consistently exhibited low binding affinities for PSGL-1.

Lin *et al.*[54] described a human SCARB2 transgenic mouse line in which the molecule is expressed ubiquitously. In these human SCRB2 transgenic mice, EV71 B genotypes are associated with HFMD-like lesions (i.e., visible hair loss and scurf) and neurological disease that include limb paralysis in 1-day-old mice but not in mice older than 2 weeks of age. In contrast, 7- and 14-day-old but not 21-day-old human SCARB2 transgenic mice are more susceptible to the C genotypes of EV71 and coxsackievirus A16, which are more likely to produce severe CNS diseases, limb paralysis and death in these mice compared to non-transgenic mice. Overall, the human SCARB2 transgenic mice have features that are similar to those of the wild-type mouse models that have previously been reported, including age-dependent susceptibility (only until 2 weeks of age), primarily viral replication sites in the muscle and CNS and pathological changes.

Fujii *et al.*[37] generated transgenic mice that express the human SCARB2 with an expression profile that is similar to that of humans (i.e., CNS neurons, lung pneumocytes, hepatocytes and intestinal epithelium). The human SCARB2 transgenic mice older than 6 weeks of age are susceptible to infection by EV71 clinical isolates and coxsackievirus A16, which use SCARB2 as a receptor after intracranial, intravenous, intraperitoneal, and intra-gastric inoculations. The mice display EV71 neurotropism, neuropathology and clinical features (i.e., ataxia, paralysis and death) that are similar to those displayed by humans, monkeys and wild-type mice. The non-neuronal tissues do not contain EV71 antigens or exhibit pathological changes, such as pulmonary edema and cutaneous lesions. However, EV71 antigens and cutaneous lesions have been noted in 1-day-old transgenic mice after infection.

Pulmonary edema and the subsequent rapid onset of cardiopulmonary failure are hallmarks of EV71-induced mortality [55]. Unfortunately, none of the mouse models, either the transgenic or non-transgenic models, exhibit pulmonary edema, although CNS neuropathology, muscular lesions, paralysis and death occur consistently following neuroinvasion. The absence of pulmonary edema limits the applicability of these murine models to the study of the disease mechanism of EV71.

Several reasons may explain why pulmonary edema is absent in EV71-infected mice. First, EV71-infected mice display a cytokine profile that is distinct from that of human patients, and the human cytokine profile may be essential for the formation of pulmonary edemas [56]. Second, the nucleus tractus solitarii (NTS), an area known to contribute to the development of pulmonary edema in rats following injury [57,58], is not the CNS target of EV71 infection in mice. Third, over-activation of the sympathetic nervous system may be required for the development of classic pulmonary edema symptoms, which may be absent in the mouse models [59].

### Animal models for testing vaccines and therapeutics

#### a. Vaccines

Both live attenuated and inactivated whole virus vaccines have achieved successful control of poliovirus [60]. Thus, it is logical to believe that EV71, which is similar to poliovirus, can also be controlled with by vaccination. The fact that passive transfer of specific antiserum provides protection against EV71 lethal challenge clearly illustrates not only the important role of humoral immunity in the control of EV71 but also the feasibility of vaccination [33]. Several EV71 vaccine candidates, including live-attenuated virus [30], inactivated whole virus [33,54,61-64], recombinant viral protein [65-67], virus-like particles and DNA vaccines [66,68,69], have been developed, and their immunogenicity and efficiency have been evaluated in animal models.

Although they are not the natural hosts for human EV71, both mice and rhesus monkeys have demonstrated the same immunogenicity (in terms of neutralization of antibody titers and specific CD8^+^ T cell responses) elicited by EV71 vaccine candidates [61]. Furthermore, it is technically feasible to assess the efficiency of inactivated vaccines in both animal species using distinct indicators, i.e., survival rates (mice), pathology (monkeys) and tissue viral loads (monkeys).

Given the age-dependent susceptibility to EV71 of laboratory mice, the EV71 vaccine candidates have primarily been tested using maternal immunization in which the dams are immunized before or during pregnancy, and the newborn pups receive lethal challenges after delivery. The efficacy of a formalin-inactivated whole-virus vaccine was first demonstrated in this setting as it prolonged the survival of pups after EV71 lethal challenge [33]. Subsequently, this strategy had been applied to the testing of other vaccine formulations [70-72].

Using an oral infection model, for the first time, we proved the concept that live vaccines are feasible for protection against EV71 [73]. In our hands, oral infection (intra-gastric challenge with mouse-adapted EV71 strains) only worked in mice that were 7 days old or younger. Despite this short time frame for immunization, we were able to demonstrate that 1-day-old mice were tolerant of an avirulent EV71 strain and mounted both systemic and mucosal antibody responses with neutralizing activities. More importantly, live EV71 active immunization at 1 day of age reduced the mortality of the mice following lethal challenge at 7 days of age. The same strategy had been applied to the testing of the efficacy of other live and inactivated EV71 vaccine candidates [54] and to examination of the therapeutic potential of a neutralizing antibody based on neonatal human SCARB2 transgenic mice [74].

Our related study showed that whole body exposures to EV71 viral suspension are an effective and less stressful alternative for inducing the lethal infection of newborn mice via the oral route [75]. In laboratory mice, fecal-oral transmission of EV71 between infected and non-infected littermates occurs after close contact [6,15]. Systemic infection of neonate rhesus monkeys has also been noted following intranasal inoculation with stool specimens from EV71-infected monkeys [9].

Non-human primates have also been used to test the immunogenicity and immunoprotection of potential EV71 vaccine candidates [32,61,68]. Macaque monkeys develop both specific humoral and cellular immune responses to both an inactivated EV71 vaccine and an EV71 virus-like particle (VLP) vaccine [68]. However, challenge experiments have not been performed to evaluate the efficacies of these vaccine candidates. Chen *et al.*[32] reported that neonatal (1.5-month-old) rhesus monkeys that are intramuscularly immunized with a formalin-inactivated, alum-based EV71 vaccine exhibit repressed viral-induced inflammatory reactions in the CNS and reduced IL-6, IFN-γ, and TNF-α production. Adult rhesus monkeys that are immunized with the inactivated vaccine also exhibit good humoral and cellular immune responses and reduced pathologies and tissue viral loads upon live virus challenge [61].

Antibody-related reactions, i.e., antibody-dependent enhanced infection [76,77] and antibody-mediated cytotoxicity [78], may represent a safety concern for vaccine development. Ch’ng *et al.*[78] demonstrated that the absence of EV71-neutralizing antibodies in the cerebrospinal fluid of vaccine-immunized and EV71-challenged monkeys, which implies that antibody-mediated responses might not be an issue, at least in monkeys.

#### b. Therapeutics

EV71 mouse models had been employed to test the anti-EV71 activities of drugs that were originally developed against human rhinoviruses and polioviruses, including pleconaril [79,80], ribavirin [34,79,81] and rupintrivir [82]. The efficacies of interferon-α [40,83] and interfering RNAs (siRNAs) [84] in inhibiting viral replication have also been examined in this setting. Generally, these anti-viral interventions exhibit certain protective or therapeutic effects when given either before or after EV71 infection that include reductions in morality and tissue virus loads and the alleviation of lesions. As susceptibility to EV71 is age dependent, only neonatal mice can be effectively infected. Thus, if an adult model were available, increasing the dosage and the number of treatments would improve the efficacy of the antiviral drugs.

Lactoferrin is a glycoprotein that is present in external secretions of mammals, such as breast milk, tears, saliva and mucous secretion [85]. Studies have demonstrated that lactoferrin can inhibit EV71 infection by blocking the absorption or receptor-mediated binding of the virus to the target cells [86,87]. Chen *et al.*[88] generated a transgenic mouse that expressed the porcine lactoferrin in milk. Interestingly, four-day-old lectoferrin transgenic mice are resistant to EV71 lethal challenge. This finding raises the possibility of developing transgenic cows or goats for the mass production of lactoferrin-enriched milks and provides an evidence base that encourages breast-feeding in humans.

## Conclusion

None of the monkey or mouse EV71 models that have been developed thus far recapitulate all of the aspects of the human disease; some of these models lack face validity (i.e., resemblance to human symptoms) and others lack construct validity (i.e., similarity to the underlying cause of the human disease). The limited studies that have been conducted in non-human primates have shown a diversity of EV71 infections in different monkey species in terms of tissue tropisms, primary viral replication sites and disease manifestations. In general, the non-human primate models are suitable for studying the course of EV71 systemic infection. Indeed, these studies using monkey models have advanced our knowledge of the transmission modes and routes, clinical manifestations, pathologies, viral distributions, and immune responses related to EV71 infection, and these factors are impossible to directly observe in human patients. Additionally, monkeys seem to be better suited than mice for evaluating the immunogenicity of EV71 vaccine candidates and are feasible for use in vaccine challenge tests that use indicators other than those used in the mouse models.

It is still unknown whether monkey EV71 neurovirulence tests will be valuable as quantitative and sensitive methods for detecting neurovirulent variants in live attenuated vaccine products because all of the EV71 genotypes and genogroups that have been tested are neurovirulent to cynomolgus monkeys. Furthermore, monkey systems would most likely be replaced by EV71 receptor-transgenic mice model when such models become available.

No adult mouse model with full validity has been established so far. Although adaptation would increase the mouse virulence of the EV71 clinical isolates, the mouse-adapted viruses themselves are unable to infect immunocompetent mice beyond weanling age. Although older immunodeficient mice (i.e., young adults) are susceptible to the adapted viruses derived from immunodeficient hosts, pulmonary edema has never been observed. Moreover, the strong myotropism of the mouse-adapted virus would definitely alter the role of virus-related neuropathology in the disease mechanism. Despite these shortcomings, neonatal mouse models with mouse-adapted viruses have contributed significantly to the study of EV71 infection [38] and vaccine development [89]. More effort should be applied to creating adult mouse models with non-mouse adapted strains. These types of model will provide better systems with longer age time frames for the induction of the disease and/or immunization.

Human PSGL-1 transgenic mice are far from practical. PSGL-1 is not expressed in neurons, which are the cellular target of EV71; thus, these transgenic mice only acquiring a transient infection of the gastrointestinal tract. Young adult human SCARB2 transgenic mice (up to 6 weeks of age) seem to be more vulnerable to the virus; however, EV71-infected mice do not express the complete set of human symptoms nor do they exhibit a disease mechanism that is similar to the underlying cause of the human disease. In theory, the identification of all of the human EV71 receptors and co-receptors and the generation of transgenic mice that express all of these receptors are crucial to EV71 research and vaccine development. The transgenic system represents a promising approach to create relevant models of EV71.

The increased size and frequency of EV71 outbreaks that have occurred over the past 16 years in the Asia-Pacific region have caused serious public health concerns. Fortunately, an inactivated EV71 vaccine has already undergone a phase III clinical trial in China [90]. Although an effective vaccine is expected in the near future, many questions regarding the nature and host response of the virus must be resolved, as these factors may compromise the safety of the vaccine. These issues include viral evolution and recombination [91,92] and antibody-mediated reactions [76-78]. Appropriate animal models with full validity are indispensable for answering these questions and providing a better system for vaccine and drug development.

## Competing interests

The authors declare that they have no competing interests.

## Authors’ contributions

YFW wrote the manuscript; CKY critically revised the manuscript. Both authors have read and approved the final manuscript.
